# Real-World Safety of Acoramidis in Transthyretin Amyloid Cardiomyopathy: A Pharmacovigilance Study

**DOI:** 10.3390/jcdd13080349

**Published:** 2026-07-26

**Authors:** Gregory W. Chai, Quynh Phi, Diego Herman Delgado

**Affiliations:** 1 Temerty Faculty of Medicine, University of Toronto, Toronto, ON M5S 3K9, Canada; 2 Schulich School of Medicine and Dentistry, University of Western Ontario, London, ON N6A 5C1, Canada; qphi@uwo.ca; 3 Division of Cardiology, Peter Munk Cardiac Centre, Toronto General Hospital, University Health Network, University of Toronto, Toronto, ON M5G 2N2, Canada; diego.delgado@uhn.ca; 4 Toronto General Hospital Research Institute, University Health Network, Toronto, ON M5G 2C4, Canada

**Keywords:** acoramidis, pharmacovigilance, transthyretin amyloidosis, FAERS, disproportionality analysis

## Abstract

Acoramidis is a novel transthyretin stabilizer recently approved for the treatment of transthyretin amyloid cardiomyopathy. Although clinical trials showed that the drug was generally safe, comprehensive real-world safety data after its approval remain limited. This study characterizes the real-world safety profile of acoramidis using the FDA Adverse Event Reporting System (FAERS) database. The database was queried for adverse events associated with acoramidis as the primary drug suspect from 1 January 2022 through 31 December 2025. Following deduplication and removal of confounding adverse events that could have stemmed from the underlying amyloidosis, a dual-statistical framework was applied. Frequentist disproportionality analysis was followed by Bayesian shrinkage using a Bayesian Confidence Propagation Neural Network to isolate statistically robust safety signals. Frequentist disproportionality analysis of 290 unique cases identified 25 safety signals. Subsequent Bayesian refinement isolated nine signals that met both frequentist and Bayesian criteria. The dual Bayesian and frequentist approaches identified signals of renal stress and gastrointestinal distress. Neuromuscular and systemic signals such as fatigue and dizziness met frequentist thresholds but did not meet the Bayesian threshold. This study’s findings reflect the previously reported gastrointestinal distress and disproportionate laboratory shifts in renal function. This study’s real-world post-marketing surveillance of acoramidis reinforces the safety data that was established through the clinical trial, ATTRibute-CM.

## 1. Introduction

Cardiomyopathy of transthyretin-mediated amyloidosis (ATTR-CM) is a progressive heart disease characterized by destabilization of the transthyretin (TTR) protein and its subsequent extracellular deposition as amyloid fibrils in the myocardium. The fibrils infiltrate, thicken, and stiffen the ventricles, contributing to diastolic dysfunction. Two forms of the disease exist: wild-type and familial. Wild-type ATTR-CM is the more common form and involves age-related deposition of normal TTR, typically in seniors above the age of 60 [1]. Familial ATTR-CM arises from genetic mutations in the TTR gene. ATTR-CM is a leading cause of restrictive cardiomyopathy and an underdiagnosed contributor of heart failure with preserved ejection fraction [1]. Stabilizer drugs have been developed to inhibit the dissociation of the tetrameric TTR protein into unstable oligomers and monomers in both wild-type and familial ATTR-CM. Tafamidis, the current first-line treatment for amyloidosis, is a stabilizer drug with long-term mortality benefits and associated lower rates of cardiovascular decline than placebo [2]. A newer stabilizer drug, acoramidis, is a small molecule drug that mimics a natural protective TTR mutation that stabilizes the protein [3]. With a mainly enthalpic binding mode involving forming hydrogen bonds, acoramidis was found to have improved binding affinity, thermodynamics, selectivity, and potency when compared to tafamidis [4]. ATTRibute-CM, the phase 3 clinical trial, has established acoramidis as a safe and effective therapy for amyloidosis, with similar rates of adverse events reported for both the treatment and placebo groups [4]. However, as acoramidis has only recently received FDA approval for the treatment of ATTR-CM, its safety profile in real-world clinical practice is yet to be established.

The risk of Adverse Drug Reactions (ADRs) may be higher in ATTR-CM patients, who tend to be older and present with multiple comorbidities treated with a wide range of medications [5,6]. As such, post-marketing surveillance in this vulnerable population is vital for the identification of rare, serious, or delayed ADRs that may not have been captured through the pre-approval clinical trials. The FDA Adverse Event Reporting (FAERS) system is a pharmacovigilance database in which patients, pharmaceutical corporations, and healthcare professionals in the United States can report adverse events (AEs). This study aims to assess FAERS data to detect real-world safety signals associated with acoramidis using disproportionality analyses, identify affected organ systems, and characterize the demographic and clinical features of reported cases, providing clinicians with more contextualized evidence that can better inform pharmacological management of ATTR-CM.

## 2. Materials and Methods

This investigation used the Food and Drug Administration’s Adverse Event Reporting System (FAERS) database to monitor the safety of acoramidis post-marketing by observing the number of AEs (https://fis.fda.gov/extensions/FPD-QDE-FAERS/FPD-QDE-FAERS.html; accessed on 16 January 2026) and did not require ethics approval as it involved analysis of de-identified, publicly accessible data. The FAERS database comprises both spontaneous and solicited individual case safety reports, including clinical-trial, expanded access and patient support program reports. As the public quarterly files do not reliably indicate report origin, no source-based filtering was applied. The FAERS data was processed using the R software, R version 4.5.2 (R Foundation for Statistical Computing, Vienna, Austria). All disproportionality metrics and the Bayesian information component were computed using custom scripts using base R and the data.table package for data management. Adverse events were coded to Preferred Terms (PTs) and grouped into System Organ Classes (SOCs) using the Medical Dictionary for Regulatory Activities (MedDRA), version 29.0, and figures were produced using the ggplot2 package. To capture all relevant reports, drug identification was performed utilizing precise regular expression (regex) boundary matching and was applied to the reported drug name (drugname) and active ingredient (prod_ai) data fields. Broad substring matching was used for the generic compound of acoramidis and its trade names (attruby and beyonttra) so all drug formulation variants including salt derivatives and free-text dosage additions entered by reporting clinicians were included. Stricter matching criteria was applied for clinical trial codes (AG10 or Ag-10) to improve specificity and prevent the inclusion of unrelated drug codes with similar character strings. This was done to ensure that only events containing the codes as standalone terms were included. These character strings were searched from 1 January 2022 to 31 December 2025. The January 2022 start date was selected on the basis of a preliminary FAERS search that identified no reports listing acoramidis in any suspect role prior to 2022. As a result, January 2022 was selected as the start point, allowing us to capture all available acoramidis reports without omission.

Data deduplication was then performed in two phases. Standard FAERS data-handling practices as detailed by Ye et al. were followed [7]. Case identifiers were used to identify unique case reports, and then the highest primary identifier for each case was retained as the most recent and updated versions of each report. The second phase involved cross-referencing the dataset with the FDA’s deleted repository, where anything that was deemed invalid or a duplicate by the FDA was also removed. Only reports where acoramidis was the primary suspect were considered in subsequent analysis.

Following that, the baseline clinical characteristics were extracted from the patient cohort. To ensure precise clinical categorization, all extracted AEs were standardized to their appropriate PTs and mapped to physiological categories using the SOC hierarchy defined by the Medical Dictionary for Regulatory Activities version 29.0. Afterwards, the SOCs were reclassified into clinically relevant groupings.

Disproportionality analyses were conducted using a dual statistical framework. Frequentist and Bayesian guidelines were consulted to validate signal robustness in accordance with the findings of Raue et al. [8] For every PT, a 2 by 2 contingency table was used to compare the observed reporting frequency to the background reporting frequency of the other drugs in the database from 2022 to 2025. Within a single report each PT was counted only once, whereas a report describing several different PTs contributed to the count for each of those terms. Cell counts were then report-based and no fractional or within-report weighting was applied. The frequentist framework was applied first. The reporting odds ratios (RORs), Proportional Reporting Ratios (PRRs) and Pearson’s chi-square statistics were calculated. Only AEs with ≥3 reports, PRR of at least 2, chi-square of at least 4, and the lower bound of the ROR 95% confidence interval (CI) greater than 1.0 were used to maintain confidence in accordance with the criteria in the findings of Evans et al. [9] As frequentist methods are susceptible to false positive inflation due to variance in rare events, the Bayesian framework was subsequently applied. The information component (IC) was calculated to measure disproportionate reporting using the Bayesian Confidence Propagation Neural Network (BCPNN) method. A signal was deemed to be present when the lower bound of the IC (IC025) was greater than 0 to eliminate signals based on high statistical variance. The IC and its approximate 95% credibility interval were derived following the standard BCPNN formulation developed at the WHO Uppsala Monitoring Centre, in which a lower credibility bound above zero (IC025 > 0) is a widely applied criterion for signalling disproportionate reporting [10,11].

Finally, to isolate AEs most likely to be attributed to physiological drug reactions, additional exclusion filters were applied. The following AEs were excluded: surgical and medical procedures, product issues related to packaging and abnormal taste, social circumstances, injury, poisoning and procedural complications. AEs potentially related to ATTR-CM were also excluded to prevent misattribution of disease progression to a drug-related reaction. These AEs included cardiac failure, cardiac failure acute and cardiac disorder. Non-events such as the improvement of pre-existing conditions were excluded as well. The excluded signals are presented in Supplemental Table S1.

## 3. Results

### 3.1. Patient Clinical Characteristics

As the dataset used for analysis only included FAERS reports from 2024–2025, and FDA approval of acoramidis occurred in 2024, the data can reasonably be expected to reflect post-marketing spontaneous reporting. A total of 290 unique adverse event reports were identified where acoramidis was found to be the primary suspect medication. The mean age of patients in the cohort was 75.7 ± 11.3 years of age. Most reports did not specify sex (87.2%, *n* = 253). Among the cases that did, male patients dominated (9.3%, *n* = 27) compared to female patients (3.4%, *n* = 10). Most reports were from the United States (81.0%, *n* = 235) geographically, followed by Japan (10.3%, *n* = 30), Europe (4.5%, *n* = 13), Germany (0.7%, *n* = 2), Belgium (0.3%, *n* = 1), and Switzerland (0.3%, *n* = 1). The remaining cases (2.8%, *n* = 8) did not specify which countries they were from. Chronologically, most of the dataset reflects post-marketing data generated after FDA approval in November of 2024. Most of the AEs were reported in 2025 (99.3%, *n* = 288). Two cases (0.7%) were from 2024. The baseline demographic and reporting characteristics of this cohort are summarized in Table 1.

### 3.2. Disproportionality Analysis

**Figure 1:**
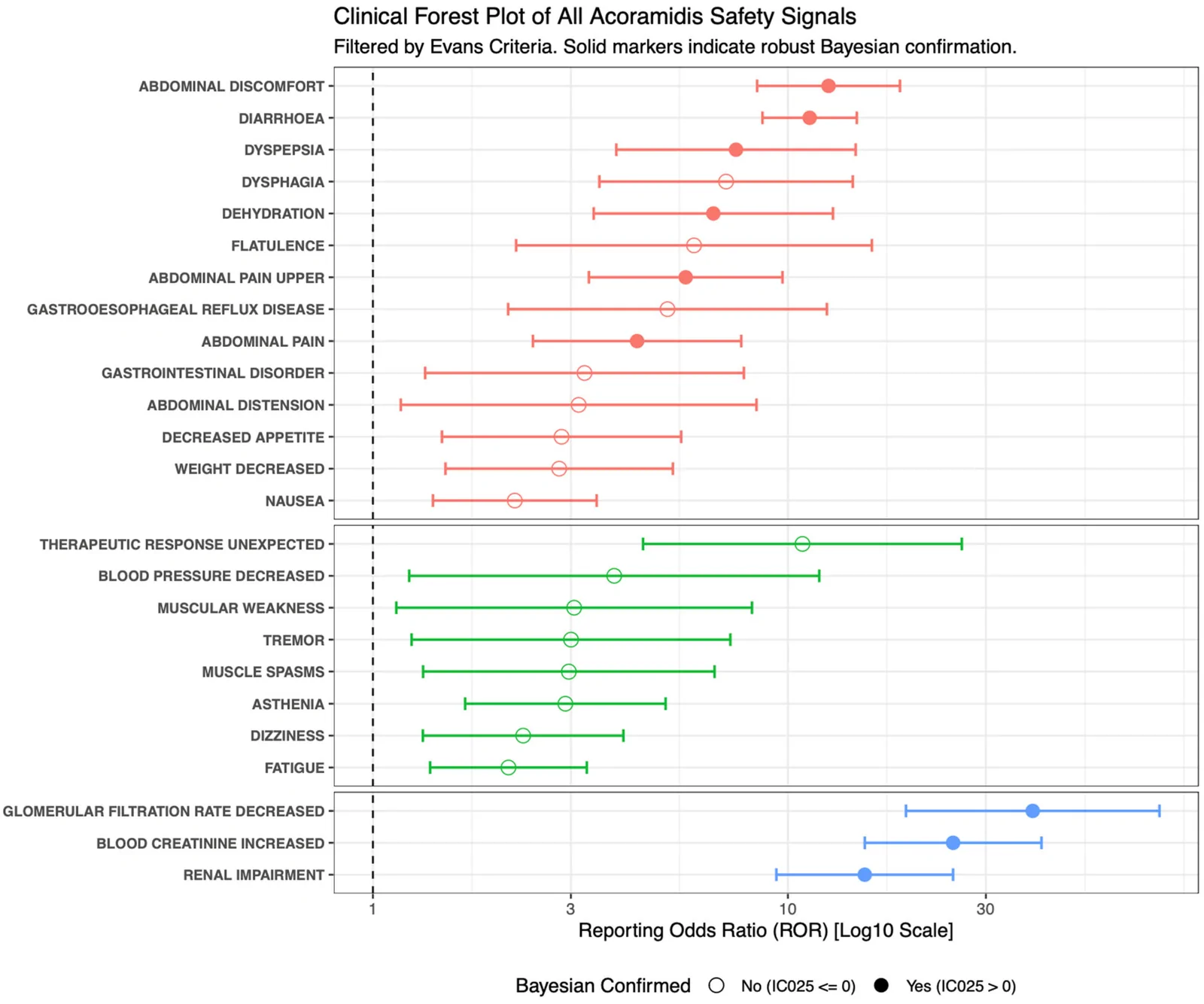
Forest plots of disproportionality signals for acoramidis cases, through reporting odds ratios and 95% confidence intervals, stratified by System Organ Class criteria while fulfilling frequentist criteria. Signals fulfilling both Bayesian and frequentist criteria were indicated using a solid circle. Signals fulfilling only frequentist criteria were indicated using a hollow circle. Gastrointestinal and nutritional signals were indicated in red. General, neurological and musculoskeletal signals were indicated in green. Renal and clearance signals were indicated in blue.

Following the exclusions of confounding signals and administrative reporting artifacts, 25 adverse event signals were identified guided by the frequentist framework. Among these, nine signals were additionally met the Bayesian threshold (IC025 > 0), indicating greater statistical stability (Figure 1; Supplemental Table S2).

### 3.3. Gastrointestinal and Nutritional Signals

Gastrointestinal disorders emerged as the most frequently reported adverse event category, consisting of 11 distinct signals. Five of these met both the frequentist and Bayesian criteria: diarrhoea (*n* = 76, ROR = 11.28, 95% CI: 8.68–14.66, PRR = 8.59, Chi-square = 525.3, IC = 3.03, IC025 = 2.05), abdominal discomfort (*n* = 27, ROR = 12.53, 95% CI: 8.43–18.62, PRR = 11.46, Chi-square = 259.6, IC = 3.27, IC025 = 1.51), upper abdominal pain (*n* = 14, ROR = 5.67, 95% CI: 3.32–9.70, PRR = 5.45, Chi-square = 51.3, IC = 2.24, IC025 = 0.46), general abdominal pain (*n* = 12, ROR = 4.33, 95% CI: 2.43–7.72, PRR = 4.19, Chi-square = 29.5, IC = 1.89, IC025 = 0.16), and dyspepsia (*n* = 9, ROR = 7.50, 95% CI: 3.86–14.56, PRR = 7.30, Chi-square = 49.1, IC = 2.45, IC025 = 0.12).

Six additional gastrointestinal signals met baseline frequentist criteria but did not meet the Bayesian threshold (IC025 ≤ 0): dysphagia (*n* = 8, ROR = 7.10, 95% CI: 3.51–14.33, PRR = 6.93, Chi-square = 40.7, IC = 2.36, IC025 = −0.04), flatulence (*n* = 4, ROR = 5.94, 95% CI: 2.21–15.94, PRR = 5.87, Chi-square = 16.2, IC = 1.93, IC025 = −0.99), gastrooesophageal reflux disease (*n* = 5, ROR = 5.13, 95% CI: 2.12–12.42, PRR = 5.06, Chi-square = 16.3, IC = 1.89, IC025 = −0.73), unspecified gastrointestinal disorder (*n* = 5, ROR = 3.24, 95% CI: 1.34–7.83, PRR = 3.20, Chi-square = 7.6, IC = 1.41, IC025 = −0.89), abdominal distension (*n* = 4, ROR = 3.13, 95% CI: 1.17–8.40, PRR = 3.10, Chi-square = 5.7, IC = 1.33, IC025 = −1.17), and nausea (*n* = 20, ROR = 2.20, 95% CI: 1.40–3.46, PRR = 2.11, Chi-square = 12.1, IC = 1.04, IC025 = −0.05).

Systemic metabolic and nutritional impacts were also present. Dehydration met both components of the dual-framework analysis (*n* = 9, ROR = 6.61, 95% CI: 3.40–12.84, PRR = 6.44, Chi-square = 41.5, IC = 2.32, IC025 = 0.08). In contrast, decreased appetite (*n* = 9, ROR = 2.85, 95% CI: 1.47–5.53, PRR = 2.79, Chi-square = 10.5, IC = 1.35, IC025 = −0.38) and weight decreased (*n* = 10, ROR = 2.81, 95% CI: 1.50–5.28, PRR = 2.75, Chi-square = 11.3, IC = 1.34, IC025 = −0.30) met only the frequentist threshold.

### 3.4. Renal and Clearance Signals

The disproportionality analysis also had a cluster of renal and clearance signals, with all three signals meeting the Bayesian threshold (IC025 > 0). These findings consist of decreased glomerular filtration rate (GFR) (*n* = 8, ROR = 38.90, 95% CI: 19.25–78.60, PRR = 37.85, Chi-square = 286.7, IC = 3.58, IC025 = 0.09), increased blood creatinine (*n* = 17, ROR = 25.01, 95% CI: 15.32–40.83, PRR = 23.60, Chi-square = 368.4, IC = 3.84, IC025 = 1.19) and renal impairment (*n* = 17, ROR = 15.32, 95% CI: 9.38–25.00, PRR = 14.48, Chi-square = 214.0, IC = 3.39, IC025 = 1.10).

### 3.5. General, Neurological and Musculoskeletal Signals

Beyond the gastrointestinal and renal systems, 10 generalized and neuromuscular signals met the baseline frequentist significance threshold, but none achieved the Bayesian threshold (all IC025 ≤ 0). General disorders included unexpected therapeutic response (*n* = 5, ROR = 10.84, 95% CI: 4.48–26.25, PRR = 10.67, Chi-square = 43.9, IC = 2.51, IC025 = −0.61), asthenia (*n* = 13, ROR = 2.91, 95% CI: 1.67–5.07, PRR = 2.82, Chi-square = 15.6, IC = 1.40, IC025 = −0.07), and fatigue (*n* = 22, ROR = 2.12, 95% CI: 1.37–3.28, PRR = 2.04, Chi-square = 12.1, IC = 0.99, IC025 = −0.04). Neurological manifestations included tremor (*n* = 5, ROR = 3.00, 95% CI: 1.24–7.27, PRR = 2.97, Chi-square = 6.6, IC = 1.33, IC025 = −0.93) and dizziness (*n* = 13, ROR = 2.30, 95% CI: 1.32–4.01, PRR = 2.24, Chi-square = 9.1, IC = 1.10, IC025 = −0.26). Musculoskeletal events highlighted muscular weakness (*n* = 4, ROR = 3.05, 95% CI: 1.14–8.19, PRR = 3.03, Chi-square = 5.5, IC = 1.30, IC025 = −1.18) and muscle spasms (*n* = 6, ROR = 2.97, 95% CI: 1.32–6.66, PRR = 2.93, Chi-square = 7.7, IC = 1.35, IC025 = −0.74). Finally, an isolated hemodynamic signal was identified for decreased blood pressure (*n* = 3, ROR = 3.81, 95% CI: 1.22–11.90, PRR = 3.79, Chi-square = 6.2, IC = 1.44, IC025 = −1.47).

## 4. Discussion

To our knowledge, this study is the first to use RORs and PRRs to analyze the AEs associated with acoramidis reported within the FAERS database. By employing a dual-framework methodology and excluding clinically confounding AEs, our analysis identified nine safety signals affecting the renal and gastrointestinal systems that were supported by both frequentist and Bayesian disproportionality analyses. These nine signals that met both criteria reflect the primary adverse reaction categorizations reported within the ATTRibute-CM clinical trial and the official FDA prescribing guidelines [12].

### 4.1. Renal Effects

The study’s findings showed that decreased GFR, increased blood creatinine, and general renal impairment were reported at a disproportionately higher frequency compared to the background rate of all other drugs in the FAERS database. This aligns with the prescribing information for acoramidis released by the FDA, which notes that acoramidis causes an increase in serum creatinine and a decrease in estimated glomerular filtration rate [12]. In the ATTRibute-CM trial cohort of patients with ATTR-CM, this manifested on Day 28 through a mean serum creatinine increase of 0.2 mg/dL and a mean eGFR decrease of 8.2 mL/min/1.73 m^2^ compared to 0.0 g/dL and 0.7 mL/min/1.73 m^2^ for the placebo [12]. A potential etiology could be that patients with advanced ATTR-CM in a heart failure state tend to experience diminished cardiac output and chronic congestion, which both compromise renal perfusion [13]. This could trigger hyperactivation of the renin-angiotensin-aldosterone system (RAAS) and sympathetic nervous systems to artificially maintain eGFR. When cardiac function improves following therapy initiation, decreases in neurohormonal hyperactivation could reveal the real, baseline eGFR through an apparent eGFR decrease. As a result, these renal signals may reflect the baseline hemodynamic fragility of patients with ATTR-CM. Importantly, the clinical trial data may support this interpretation, noting that these laboratory shifts generally occur within 4 weeks of starting therapy, stabilize over time, and are fully reversible following treatment discontinuation [12]. This is analogous to RAAS inhibitors such as angiotensin-converting enzyme inhibitors and angiotensin receptor blockers, where a mild and non-progressive drop in eGFR early after treatment initiation is expected and acceptable [14]. However, it is crucial to note that causality cannot be established from spontaneous reporting data and that these renal signals may instead reflect a potential direct nephrotoxic mechanism of acoramidis itself. Further studies are needed to conclusively determine the true source of the observed effects on the kidney and renal function.

Interestingly, neither the clinical trial nor the post-marketing pharmacovigilance studies for tafamadis indicated any drug-related kidney effects, unlike acoramidis. However, this could be explained by looking at the mechanism of action of both stabilizer drugs. As acoramidis is able to achieve a superior near-complete stabilization of the tetramer as opposed to tafamidis’ less rapid and uniform effect, acoramidis alone may yield a faster onset of cardiac improvement and changes in renal hemodynamics [15]. The kidneys act as a primary clearance pathway for numerous pharmacological agents. As such, clinicians initiating acoramidis in patients with borderline baseline renal function must maintain longitudinal monitoring of GFR and creatinine to remain attentive against cardiorenal syndrome.

### 4.2. Gastrointestinal and Systemic Tolerability

Gastrointestinal AEs had the greatest number of signals, consisting of signals that met the Bayesian threshold, such as diarrhoea, abdominal discomfort, upper abdominal pain, general abdominal, dehydration and dyspepsia. Eight additional GI signals met the baseline frequentist criteria but did not meet the Bayesian threshold.

This study provides a profile of gastrointestinal AEs associated with acoramidis in a real clinical setting. These results validate the ATTRibute-CM data, which highlights GI adverse reactions as the primary clinical side effect of acoramidis therapy [12]. The trial noted elevated rates of diarrhea (11.6% versus 7.6% for placebo) and upper abdominal pain (5.5% versus 1.4% for placebo) [12]. This GI signal may be the result of a two-hit mechanism. The localized enteric irritation of oral acoramidis may act as an acute trigger on a gastrointestinal tract already rendered neurologically vulnerable by amyloid-induced autonomic neuropathy or mucosal deposition, which leads to malabsorption or protein-losing enteropathy [16]. However, this is mainly in hereditary ATTR amyloidosis instead of wild-type ATTR-CM [17]. As it remains unclear whether GI symptoms are caused by the amyloidosis, the drug, or another unrelated entity, further research is needed.

The clinical trial data notes that the majority of these GI adverse reactions are categorized as mild and generally resolve without requiring drug discontinuation. However, in a fragile senior population with heart complications, unmanaged GI distress and subsequent dehydration and weight loss can pose risks. In these patients who may also take diuretics, dehydration can exacerbate cardiorenal syndrome, where impaired compensatory mechanisms cause fluid depletion with diuretic use, leading to inadequate maintenance of blood pressure and an increased risk of acute kidney injury [18]. The chronic burden of these symptoms also impairs quality of life and medication adherence, which is essential in a likely polypharmacy-managed population [19].

### 4.3. Neuromuscular and Systemic Signals

Beyond the gastrointestinal and metabolic clusters, the frequentist analysis flagged several generalized, neurological, and musculoskeletal signals, none of which met the Bayesian threshold. Notably, none of these specific eight signals are classified as primary adverse reactions in the ATTRibute-CM trial [12]. This validates our dual-statistical methodology: while the more sensitive frequentist equations flagged these events as potential signals, the more specific Bayesian neural network identified them as less robust, aligning with the results of the trial. The failure to meet the Bayesian threshold and the lack of identification within the trial suggests that these signals are unlikely to reflect drug-induced events. Instead, these findings may be more plausibly explained by the clinical profile of ATTR cardiomyopathy and subsequent heart failure. For example, patients with ATTR cardiomyopathy commonly present with fatigue, a symptom of associated heart failure [20]. Hypotension was also documented, which is likely due to being treated with heart failure medications such as β-blockers and angiotensin receptor blockers [20]. Interestingly, tremors and muscle spasms are not recognized as typical manifestations of ATTR amyloidosis, highlighting the need for further exploration in future studies.

### 4.4. Strengths and Limitations

This study utilizes a complex methodology employed by other authors in the pharmacovigilance field in order to address multiple aspects of data collection and analysis. Using both frequentist sensitivity and Bayesian specificity allows for greater filtering of false positives and the most stable signals out of the initial 25. However, this study is also subject to the inherent limitations of spontaneous reporting systems, including underreporting, missing information, and low-quality data. Notably, as the platform does not facilitate identification of the total number of patients taking the drug and relies on voluntary reports from patients and consumers, true incidence rates are not captured. As a result, this study cannot determine the absolute risk of an AE. Though the use of disproportionality metrics (ROR, PRR) and Bayesian methods (IC) aimed to accurately capture relative risk, these metrics still used the background reporting rate of other drugs, which can be affected by the composition of the database. Specifically, since the patients with ATTR-CM tend to be older and more heavily co-medicated than the general FAERS population, these disproportionality estimates may be inflated by indication bias, confounding by disease severity, and age-related reporting patterns rather than reflecting a true drug effect. Lastly, due to relatively small case numbers, many signals have limited stability. Therefore, these findings should be seen as hypothesis-generating and interpreted with caution. As this drug has only recently been approved for clinical use, future studies with larger sample sizes can be conducted once more post-marketing FAERS data becomes available.

## 5. Conclusions

In this real-world pharmacovigilance analysis, acoramidis demonstrates a safety profile consistent with clinical trial data, characterized predominantly by gastrointestinal adverse events and laboratory markers of renal function change. The observed renal signals may reflect hemodynamic adaptations rather than intrinsic nephrotoxicity, though causality cannot be established from spontaneous reporting data. Careful monitoring remains warranted in a vulnerable heart failure patients. As the therapeutic landscape for ATTR-CM expands, recognizing these systemic signals will be vital for optimizing patient tolerability, managing nutritional status, and navigating the delicate cardio-renal balance in this complex clinical population.

## Figures and Tables

**Table 1:** Baseline demographic and reporting characteristics of acoramidis primary suspect cases.

Characteristic	*n* (%)
Total Events (N)	290
Mean Age (Years) ± SD	75.7 ± 11.3
Sex	
Male	27 (9.3)
Female	10 (3.4)
Not-specified	253 (87.2)
Reporting Location	
United States	235 (81.0)
Japan	30 (10.3)
Europe	13 (4.5)
Germany	2 (0.7)
Belgium	1 (0.3)
Switzerland	1 (0.3)
Not Specified	8 (2.8)
Reporting Year	
2024	2 (0.7)
2025	288 (99.3)

## Data Availability

All data relevant to this study are included in the article, and any additional data may be obtained from the corresponding author on reasonable request. The dataset analyzed for this current study is available through the FDA Adverse Event Reporting System (FAERS) database.

## Supplementary Materials

The following supporting information can be downloaded at https://www.mdpi.com/article/10.3390/jcdd13080349/s1, Table S1: Excluded signals PT (Preferred Term) from acoramidis adverse event analysis with the number of primary suspect cases, reporting odds ratio (ROR), 95% confidence interval (CI) and information component 25 (IC025) were included; Table S2: Comprehensive Frequentist and Bayesian Disproportionality Statistics consisting of reporting odds ratio, Proportional Reporting Ratio, Chi-Squared value, and BCPNN information component of Acoramidis adverse events that fulfilled Evan’s Criteria.

## Abbreviations

The following abbreviations are used in this manuscript:

ADR	Adverse Drug Reaction
AEs	Adverse events
ATTR	Transthyretin amyloidosis
ATTR-CM	Transthyretin amyloid cardiomyopathy
BCPNN	Bayesian Confidence Propagation Neural Network
CI	Confidence interval
FAERS	Food and Drug Administration Adverse Event Reporting System
GFR	Glomerular filtration rate
GI	Gastrointestinal
IC	Information component
PRR	Proportional Reporting Ratio
PT	Preferred Term
RAAS	Renin-angiotensin-aldosterone-system
ROR	Reporting odds ratio
SOC	System Organ Class
TTR	Transthyretin
