# Supplementary material for: Real-World Safety of Acoramidis in Transthyretin Amyloid Cardiomyopathy: A Pharmacovigilance Study
Source: J Cardiovasc Dev Dis. 2026 Jul 26;13(8):349. doi: 10.3390/jcdd13080349 (PMC13513231; doi:10.3390/jcdd13080349)
Supplement: Supplementary file 1 [file jcdd-13-00349-s001.zip › jcdd-4400558-supplementary.pdf]

Supplemental Table S1. Excluded signals PT (preferred term) from acoramidis adverse event analysis with number of primary suspect cases, reporting odds ratio (ROR), 95% confidence interval (CI) and the information component 25 (IC025) were included.

PT	N (Primary Suspect Cases)	ROR	ROR (95% CI)	IC025
CARDIAC DISORDER	2	2.03	(0.51 - 8.18)	-2.18
CARDIAC FAILURE	2	2.07	(0.51 - 8.31)	-2.17
CARDIAC FAILURE ACUTE	1	10.2	(1.43 - 72.66)	-3
PRE-EXISTING CONDITION IMPROVED	1	22.36	(3.14 - 159.45)	-3.01
PRODUCT DOSE OMISSION ISSUE	13	1.46	(0.84 - 2.54)	-0.7
WRONG TECHNIQUE IN PRODUCT USAGE PROCESS	6	1.67	(0.74 - 3.74)	-1.13
PRODUCT STORAGE ERROR	5	2.08	(0.86 - 5.05)	-1.13
INTENTIONAL PRODUCT USE ISSUE	3	1.61	(0.52 - 5.03)	-1.82
PRODUCT USE ISSUE	3	1.07	(0.34 - 3.33)	-2.09
INAPPROPRIATE SCHEDULE OF PRODUCT ADMINISTRATION	2	0.42	(0.11 - 1.70)	-3.23
INTENTIONAL DOSE OMISSION	2	2.42	(0.60 - 9.73)	-2.12
UNDERDOSE	2	2.34	(0.58 - 9.42)	-2.13
ARTHROPOD BITE	1	10.52	(1.48 - 75.00)	-3
BACK INJURY	1	6.02	(0.84 - 42.87)	-2.99
BONE CONTUSION	1	53.68	(7.52 - 383.38)	-3.02
BRAIN CONTUSION	1	170.68	(23.76 - 1225.99)	-3.03
CERVICAL VERTEBRAL FRACTURE	1	24.47	(3.43 - 174.51)	-3.01

CIRCUMSTANCE OR INFORMATION CAPABLE OF LEADING TO MEDICATION ERROR	1	1.25	(0.17 - 8.87)	-3.18
CONTUSION	1	0.81	(0.11 - 5.77)	-3.36
EXPOSURE TO EXTREME TEMPERATURE	1	256.02	(35.49 - 1846.67)	-3.03
FALL	1	0.25	(0.03 - 1.77)	-4.24
OFF LABEL USE	1	0.05	(0.01 - 0.37)	-6.04
PRESCRIBED UNDERDOSE	1	3.02	(0.42 - 21.55)	-3.01
PRODUCT DOSE OMISSION IN ERROR	1	1.01	(0.14 - 7.18)	-3.26
SKIN WOUND	1	30.67	(4.30 - 218.82)	-3.02
PRODUCT PACKAGING QUANTITY ISSUE	5	21.3	(8.79 - 51.59)	-0.61
PRODUCT PACKAGING DIFFICULT TO OPEN	3	45.94	(14.71 - 143.45)	-1.42
PRODUCT PACKAGING ISSUE	2	15.53	(3.86 - 62.44)	-2
PRODUCT SIZE ISSUE	2	26.22	(6.52 - 105.45)	-2.01
PRODUCT LABEL ISSUE	1	10.17	(1.43 - 72.52)	-3
PRODUCT SUPPLY ISSUE	1	5.12	(0.72 - 36.48)	-2.99
PRODUCT TEMPERATURE EXCURSION ISSUE	1	5.74	(0.81 - 40.89)	-2.99
INABILITY TO AFFORD MEDICATION	1	4	(0.56 - 28.52)	-3
THERAPY CESSATION	3	4.14	(1.33 - 12.90)	-1.46
COLON OPERATION	1	56.57	(7.92 - 404.07)	-3.02
DIALYSIS	1	8.29	(1.16 - 59.07)	-2.99
HAEMODIALYSIS	1	49.92	(6.99 - 356.48)	-3.02

THERAPY CHANGE	1	5.63	(0.79 - 40.15)	-2.99
THERAPY INTERRUPTED	1	0.43	(0.06 - 3.06)	-3.77

PT: Preferred Term

ROR: Reporting Odds Ratio

CI: Confidence Interval

IC: Information Component

Supplemental Table S2. Comprehensive Frequentist and Bayesian Disproportionality Statistics consisting of Reporting Odds Ratio, Proportional Reporting Ratio, Chi-Squared value, and BCPNN Information Component of Acoramidis Adverse Events that fulfilled Evan's Criteria

PT	N	ROR	ROR (95% CI)	PRR	Chi-Sq	IC	IC025
GLOMERULAR FILTRATION RATE DECREASED	8	38.9	(19.25 - 78.60)	37.85	286.7	3.58	0.09
BLOOD CREATININE INCREASED	17	25.01	(15.32 - 40.83)	23.6	368.4	3.84	1.19
RENAL IMPAIRMENT	17	15.32	(9.38 - 25.00)	14.48	214	3.39	1.1
ABDOMINAL DISCOMFORT	27	12.53	(8.43 - 18.62)	11.46	259.6	3.27	1.51
DIARRHOEA	76	11.28	(8.68 - 14.66)	8.59	525.3	3.03	2.05
THERAPEUTIC RESPONSE UNEXPECTED	5	10.84	(4.48 - 26.25)	10.67	43.9	2.51	-0.61
DYSPEPSIA	9	7.5	(3.86 - 14.56)	7.3	49.1	2.45	0.12
DYSPHAGIA	8	7.1	(3.51 - 14.33)	6.93	40.7	2.36	-0.04
DEHYDRATION	9	6.61	(3.40 - 12.84)	6.44	41.5	2.32	0.08
FLATULENCE	4	5.94	(2.21 - 15.94)	5.87	16.2	1.93	-0.99
ABDOMINAL PAIN UPPER	14	5.67	(3.32 - 9.70)	5.45	51.3	2.24	0.46
GASTROOESOPHAGEAL REFLUX DISEASE	5	5.13	(2.12 - 12.42)	5.06	16.3	1.89	-0.73
ABDOMINAL PAIN	12	4.33	(2.43 - 7.72)	4.19	29.5	1.89	0.16
BLOOD PRESSURE DECREASED	3	3.81	(1.22 - 11.90)	3.79	6.2	1.44	-1.47
GASTROINTESTINAL DISORDER	5	3.24	(1.34 - 7.83)	3.2	7.6	1.41	-0.89
ABDOMINAL DISTENSION	4	3.13	(1.17 - 8.40)	3.1	5.7	1.33	-1.17
MUSCULAR WEAKNESS	4	3.05	(1.14 - 8.19)	3.03	5.5	1.3	-1.18

TREMOR	5	3	(1.24 - 7.27)	2.97	6.6	1.33	-0.93
MUSCLE SPASMS	6	2.97	(1.32 - 6.66)	2.93	7.7	1.35	-0.74
ASTHENIA	13	2.91	(1.67 - 5.07)	2.82	15.6	1.4	-0.07
DECREASED APPETITE	9	2.85	(1.47 - 5.53)	2.79	10.5	1.35	-0.38
WEIGHT DECREASED	10	2.81	(1.50 - 5.28)	2.75	11.3	1.34	-0.3
DIZZINESS	13	2.3	(1.32 - 4.01)	2.24	9.1	1.1	-0.26
NAUSEA	20	2.2	(1.40 - 3.46)	2.11	12.1	1.04	-0.05
FATIGUE	22	2.12	(1.37 - 3.28)	2.04	12.1	0.99	-0.04

PT: Preferred Term

ROR: Reporting Odds Ratio

PRR: Proportional Reporting Ratio

CI: Confidence Interval

IC: Information Component
